# Laser-Interferometric Broadband Seismometer for Epicenter Location Estimation

**DOI:** 10.3390/s17102423

**Published:** 2017-10-23

**Authors:** Kyunghyun Lee, Hyungkwan Kwon, Kwanho You

**Affiliations:** Department of Electrical and Computer Engineering, Sungkyunkwan University, Suwon 16419, Korea; naman2001@skku.edu (K.L.); ant6565@naver.com (H.K.)

**Keywords:** seismic wave, laser interferometer, epicenter localization, STA/LTA, range difference of arrival

## Abstract

In this paper, we suggest a seismic signal measurement system that uses a laser interferometer. The heterodyne laser interferometer is used as a seismometer due to its high accuracy and robustness. Seismic data measured by the laser interferometer is used to analyze crucial earthquake characteristics. To measure P-S time more precisely, the short time Fourier transform and instantaneous frequency estimation methods are applied to the intensity signal ($I_{y}$) of the laser interferometer. To estimate the epicenter location, the range difference of arrival algorithm is applied with the P-S time result. The linear matrix equation of the epicenter localization can be derived using P-S time data obtained from more than three observatories. We prove the performance of the proposed algorithm through simulation and experimental results.

## 1. Introduction

The fundamental reasons for earthquakes arise from natural causes and artificial explosions. Natural causes by the movement of tectonic plates bring about energy spouting from the earth’s interior, and volcano eruption. Artificial earthquakes are generated by explosions, collapses of large buildings, etc. A seismometer records the vibration caused by natural and artificial earthquakes. Many researchers have used the seismic data measured by seismometer to investigate the features of an earthquake, such as its magnitude, epicenter, and crust pattern. However, despite comprehensive earthquake study, the prediction of earthquake is very difficult since the measurement of seismic wave contains the noise factor during the measurement process [1,2,3,4]. The measurement noise occurs inevitably by the imperfection of the measurement environment and the background seismic signals. To investigate earthquake prediction, a precision instrument is needed to measure micro-earthquakes [5,6,7]. Usually, micro-earthquakes are generated prior to and subsequent to a strong earthquake, and they are termed foreshocks and aftershocks [8], respectively. Therefore, the study of micro-earthquakes is important in seismology. In particular, if a foreshock can be precisely estimated, the damage from a strong earthquake may be lessened.

There have been many efforts to precisely detect and analyze earthquakes. Beyreuther [9] suggested a method to detect and classify earthquakes using hidden Markov modeling, instead of a short-term average over a long-term average (STA/LTA) detector. Botella [10] proposed a real-time earthquake detector with pre-filtering by wavelets. The use of a discrete wavelet transform could increase the detector reliability. Araya [11] suggested a highly sensitive wideband seismometer using a Michelson laser interferometer. This made more exact measurement of the seismic wave than other devices under the noise environment. The epicenter location is very difficult to obtain exactly in spite of its importance [12,13,14]. Zhu [14] proposed a new estimation algorithm for epicenter location using low frequency seismograms. This is faster than the time domain method, and can also compensate the error caused by low sampling rate. Gasparini [15] suggested a real-time earthquake location technique for early warning based on an equal differential time formulation and a probabilistic approach for hypocenter estimation.

In this paper, we propose a precision seismometer system with a laser interferometer [16,17,18,19]. Contrary to the seismometer based on accelerometers, it can determine the arrival time of a P-S wave and the epicenter location more accurately. The measured seismic wave ($I_{y}$) by the heterodyne laser interferometer in the time domain is converted into the time-frequency domain with short-time Fourier transform (STFT) [20,21,22,23] and instantaneous frequency (IF) estimation as preprocessing [24,25]. The STFT is a useful tool to analyze non-stationary signals and time-varying systems. In order to more exactly examine the seismic signal with time changes, the IF estimation method is applied to the incoming signal sequences. IF estimation can extract the frequency variation of a seismic wave with time changes. The data in the time-frequency domain is applied and formulated as an STA/LTA ratio to calculate the distance from the epicenter.

In general, the triangulation method is applied to estimate the location of epicenter using three epicentral distances. The triangulation method has weakness about estimation accuracy since it estimates the epicenter location without considering measurement noise. To find the epicenter, the range difference of arrival (RDOA) algorithm [26,27,28,29] is used. RDOA method represents the problem in a linear matrix equation and facilitates to apply many kinds of optimization methods. Moreover, the RDOA method has an advantage with the low computational complexity. With the difference of distances that are measured from at least 3 observatories, the origin of the signal spread can be estimated as the epicenter.

This paper is organized as follows. Section 2 describes the seismic wave measurement system with a heterodyne laser interferometer. Section 3 explains P-S time detection with the STFT, IF and STA/LTA method. Section 4 determines the epicenter location with the RDOA algorithm. Section 5 demonstrates the effectiveness of the proposed algorithm through the simulation results, while Section 6 concludes the paper.

## 2. Heterodyne Laser Interferometer

**Figure 1:**
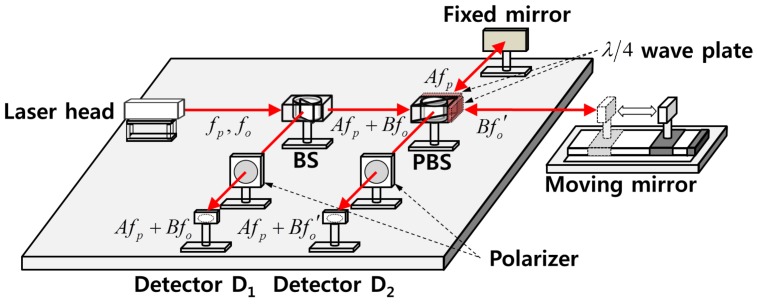
Schematic diagram of seismic wave measurement system using a laser interferometer.

The measurement of seismic wave is a significant problem since the measurement accuracy is related directly with the accuracy of P-S time computation. In this paper, a heterodyne laser interferometer as a high precision displacement measurement instrument is used for the seismic wave measurement. A heterodyne laser interferometer measures displacement using the orthogonal characteristic of a laser source [30,31,32,33]. The laser head emits two polarized beams that have different frequencies. The laser source is divided at the beam splitter (BS) equally into two beams that have the same form. One moves downwards to the detector $D_{1}$, while the other proceeds towards the polarized beam splitter (PBS). The laser sources emitted from the PBS are separated again into two beams. After being reflected by a moving mirror and a fixed mirror respectively, the separated beams are recombined, and collected at the detector $D_{2}$. Figure 1 shows a schematic diagram of seismic detection system based on the heterodyne laser interferometer. The measurement part of seismic detection system needs to be isolated from the seismic wave’s effect using the vibration isolation system [34].

The electric fields that are collected at detector $D_{1}$ are represented as follows [35]:

(1) $$\begin{array}{ccc} E_{Ap} & = & \frac{1}{\sqrt{2}}Ae^{j(2\pi f_{p}t+\phi_{A})},\\ E_{Ao} & = & \frac{1}{\sqrt{2}}Be^{j(2\pi f_{o}t+\phi_{B})}, \end{array}$$

where *A* and *B* are the amplitudes of the electric field, and $\phi_{A}$, $\phi_{B}$ are the initial phase values of the given electric field. The laser head emits two-frequency beams of $f_{p}$ for vertical polarization and $f_{o}$ for horizontal polarization, respectively. The electric fields measured at detector $D_{2}$ are expressed as follows:

(2) $$\begin{array}{ccc} E_{Bp} & = & \frac{1}{\sqrt{2}}Ae^{j(2\pi f_{p}t+\phi_{A})},\\ E_{Bo} & = & \frac{1}{\sqrt{2}}Be^{j(2\pi f_{o}t+\phi_{B}+\Delta \phi )}, \end{array}$$

where Δϕ is the phase difference occurred by the Doppler effect: $\Delta \phi =2\pi (f_{o}^{\prime }-f_{o})t$. $f_{p}$ and $f_{o}$ are the frequencies of the two orthogonal polarized beams from a laser head. The intensities of the reference signal and measurement signal that are collected by photo detectors $D_{1}$ and $D_{2}$ are represented as:

(3) $$\begin{array}{cc} I_{R} & \propto \;\left(E_{Ap}+E_{Ao}\right){\left(E_{Ap}+E_{Ao}\right)}^{*}\\ & =\;\frac{1}{2}\left(A^{2}+B^{2}\right)+AB\cos\left[2\pi \Delta ft+(\phi_{B}-\phi_{A})\right],\\ I_{M} & \propto \;\left(E_{Bp}+E_{Bo}\right){\left(E_{Bp}+E_{Bo}\right)}^{*}\\ & =\;\frac{1}{2}\left(A^{2}+B^{2}\right)+AB\cos\left[2\pi \Delta ft+(\phi_{B}-\phi_{A})+\Delta \phi \right]. \end{array}$$

where Δf means the frequency difference of $f_{p}-f_{o}$. The DC components of the measured intensity signals are removed through a high-pass filter. The remaining signals are entered into a lock-in amplifier to obtain the phase value. Then, after passing through a lowpass filter, the intensities of $I_{x}\propto I_{M,ac}\cdot I_{R}$ and $I_{y}\propto I_{M,ac}\cdot I_{R}e^{j\pi /2}$ are represented as [36]:

(4) $$\begin{array}{ccc} I_{x} & \propto & \frac{AB}{2}\cos\left(\Delta \phi \right),\\ I_{y} & \propto & \frac{AB}{2}\sin\left(\Delta \phi \right). \end{array}$$

The phase value can then be extracted by solving the trigonometric Equation (4). Moreover, the moving object’s displacement can be calculated from the relation between the optical length and the phase value.

## 3. P-S Time Detection With Instantaneous Frequency

Generally, seismic data that are detected by a seismometer are analyzed in the time-amplitude domain. When an earthquake happens, the seismic signals are measured by observation of the P-wave and S-wave. The P-S time is determined according to the difference of velocity and amplitude between the P-wave and S-wave. However, if the difference of amplitude between the P-wave and S-wave is too small, the measurement of P-S time becomes difficult to calculate. To overcome this problem, we analyze the seismic wave in the time-frequency domain. In order to represent seismic data in the frequency domain, the STFT algorithm is applied. When the seismic signal is transformed into the frequency domain through the STFT, the STFT result represents the frequency change and amplitude value of the seismic signal in frequency with time. Hence, the P-S time is obtained by using the STFT.

With the displacement measurement by the heterodyne laser interferometer, the phase value can be interpreted as proportional to the displacement, as follows:

(5) $$\begin{array}{c} \Delta \phi =\frac{4\pi nD}{\lambda_{m}}, \end{array}$$

where *n* is an air refractive index, and $\lambda_{m}$ represents the mean of wavelengths of a laser source. *D* is the displacement, and Δϕ is obtained from Equation (4). The seismic data that are measured by the laser interferometer can be represented as a trigonometric function with the phase information:

(6) $$\begin{array}{ccc} {\hat{I}}_{x} & \propto & \frac{AB}{2}\cos\left(\Delta \hat{\phi }\right),\\ {\hat{I}}_{y} & \propto & \frac{AB}{2}\sin\left(\Delta \hat{\phi }\right), \end{array}$$

where ${\hat{I}}_{x}$, ${\hat{I}}_{y}$, and $\Delta \hat{\phi }$ are the measurement values, respectively. With no loss of generality, we use the measured intensity signal (${\hat{I}}_{y}$) as a seismic data.

Hence, the amplitude change of a seismic signal can be detected with the change of frequency. STFT algorithm is used to represent the detected signal in the time-frequency domain. Fourier transform enables the conversion of a function in the time domain to the frequency domain. However, when data in the time domain is transformed into the frequency domain by the Fourier transform, the time information of the transformed data is lost, and represented in terms of the frequency and amplitude, as the data is calculated for the whole time-interval. The Fourier transform is a function of the angular frequency ω. Therefore, it is impossible to obtain the time-frequency data. To analyze the seismic data in the time-frequency domain, we apply the short-time Fourier transform, which is a modification of the Fourier transform, and has short-term sampling intervals. The STFT of f(t) is defined as follows [37,38]:

(7) $$\begin{array}{c} STFT\left\{f(t)\right\}\left(\tau ,\;\omega \right)=\int_{-\infty }^{\infty }f\left(t\right)h\left(t-\tau \right)e^{-j\omega t}dt, \end{array}$$

where h(t) is a window function for analysis. The shape and the size of the window function affect the resolution of the STFT. When the STFT is applied, the transformed data is represented in time-frequency division as a spectrum, because the STFT is based on the Fourier transform in each time interval. Therefore, we can show the frequency change of the given data as a frequency spectrum. In the STFT method, it is hard to represent the transformed seismic signal in a closed form. To analyze the data as a single value in the time-frequency domain, we use the instantaneous frequency estimation method.

The instantaneous frequency is defined as the derivative of a phase. Generally, the instantaneous frequency is obtained as a single value, by using methods such as counting zero-crossing, and phase differentiation. Other methods for instantaneous frequency estimation are time-frequency distribution with the STFT, wavelet transform, and S-transform. Therefore, the STFT is used for instantaneous frequency estimation to analyze the data in the time-frequency domain. In the time-frequency distribution by the STFT, a single frequency value on time is obtained by an instantaneous frequency estimation. The instantaneous frequency estimation X(t) is defined as follows [39,40]:

(8) $$\begin{array}{c} X\left(t\right)=\arg\left[\max TFD(t,f)\right], \end{array}$$

where TFD(t,f) is the time-frequency distribution of a seismic signal that is applied to the STFT. Then, the obtained data is represented in the time-frequency domain, and the arrival time of the P-wave and S-wave can be determined.

STA/LTA is one of the most frequently used methods in seismology to find the P-S time. To determine the arrival time of the P and S wave, the steepness of change rate is an important indicator. STA/LTA uses two moving windows which have different sizes. One has a short-sized window, and the other has a long-sized window. The short-sized moving window is more sensitive to the change of a seismic signal. The long-sized window has a gentle slope. With the characteristic of each window, the variation of seismic data can be detected. The short-term average and the long-term average [41,42] are represented as follows:

(9) $$\begin{array}{ccc} A_{S}\left(k\right) & = & \sum_{t=k-n_{s}}^{k}\frac{X(t)}{n_{s}},\\ A_{L}\left(k\right) & = & \sum_{t=k-n_{l}}^{k}\frac{X(t)}{n_{l}}, \end{array}$$

$n_{s}$ and $n_{l}$ are the short-term window size, and the long-term window size, respectively. Finally, to determine the arrival time of the P-wave and S-wave, we set the threshold value. If $v_{p}$, $v_{s}$ are defined as the velocities of the P-wave and S-wave, respectively, and $t_{ps}$ is the P-S time that is obtained by STA/LTA algorithm, the distance ($D_{e}$) from an observatory to the epicenter can be measured by using Equation (10) as follows:

(10) $$\begin{array}{c} D_{e}=\frac{v_{p}v_{s}}{v_{p}-v_{s}}t_{ps}. \end{array}$$

## 4. Epicenter Localization Based on Range Difference of Arrival

In this section, the RDOA method is used to determine the location of an epicenter. Although the triangulation method has been mostly used for epicenter localization due to its simplicity, there exists a limited accuracy problem caused by not considering the measurement noise. The RDOA method derives the relatively precise location of epicenter with the low computational complexity. The RDOA is the application of the time difference of arrival (TDOA) [43,44,45]. The TDOA uses the different reaching time from emitter to receiver, and it can be transformed to the RDOA, by multiplying the propagation velocity. The TDOA equation is expressed as follows:

(11) $$\begin{array}{c} t_{ij}=t_{i}-t_{j},\;\;i,j\in \left\{1,2,\cdots ,m\right\}, \end{array}$$

where $t_{i}$ is a propagation time, and *m* is the number of receivers that are considered as observatories. According to the relation between distance and time, the RDOA equation can be expressed as follows:

(12) $$\begin{array}{c} s_{i}^{o}=t_{i}\times v_{sig}, \end{array}$$

where $s_{i}^{o}$ is the distance from the epicenter to each observatory, and $v_{sig}$ is the propagation velocity of the seismic wave.

To determine the epicenter location, we need at least three earthquake observatories. Following the definition of a hyperbola equation, we set the observatory locations as focal points of each hyperbola, and find the point of intersection after depicting two pairs or more hyperbolae. Let us assume that there are *m* observatories that use the laser seismometer to detect a seismic wave. The measured seismic signals and the arrival time in each observatory are different, because the distances from the epicenter to each observatory are not the same.

RDOA is one of the popular location decision methods. We apply RDOA algorithm based on the least square method. The location of the *i*-th observatory is defined as $o_{i}={\left[x_{i}\;y_{i}\right]}^{T}$, i=1,2,⋯,m. The epicenter location is set as ζ = ${\left[x\;y\right]}^{T}$. We set the RDOA measurement value as:

(13) $$\begin{array}{ccc} s_{i}^{o} & = & ∥\zeta -o_{i}∥,\\ s_{i1}^{o} & = & s_{i}^{o}-s_{1}^{o}, \end{array}$$

where $s_{i}^{o}$ is the distance between the epicenter and the *i*-th observatory. $s_{i1}^{o}$ is the RDOA value between the *i*-th observatory and the first observatory. Using $s_{i}^{o}=∥\zeta -o_{i}∥$, the square of the *i*-th observatory distance from the epicenter can be written as:

(14) $$\begin{array}{cc} {\left(s_{i}^{o}\right)}^{2} & =\;\langle \zeta -o_{i},\zeta -o_{i}\rangle \\ & =\;{∥\zeta ∥}^{2}-2o_{i}^{T}\zeta +{∥o_{i}∥}^{2}. \end{array}$$

Equation (14) can be rewritten as follows:

(15) $$\begin{array}{cc} {\left(s_{i}^{o}\right)}^{2} & =\;{\left(s_{i1}^{o}+s_{1}^{o}\right)}^{2}\\ & =\;\langle \zeta -o_{1},\zeta -o_{1}\rangle +2s_{i1}^{o}s_{1}^{o}+{\left(s_{i1}^{o}\right)}^{2}. \end{array}$$

As Equation (14) is equivalent to Equation (15), we represent the result as a quadratic form:

(16) $$\begin{array}{c} {∥\zeta ∥}^{2}-2o_{1}^{T}\zeta +∥o_{1}{∥}^{2}+2s_{1}^{o}s_{i1}^{o}+{\left(s_{i1}^{o}\right)}^{2}={∥\zeta ∥}^{2}-2o_{i}^{T}\zeta +{∥o_{i}∥}^{2}. \end{array}$$

Then the relation among observatories can be formulated as

(17) $$\begin{array}{c} ∥o_{1}{∥}^{2}-{∥o_{i}∥}^{2}+{\left(s_{i1}^{o}\right)}^{2}=2\langle o_{1}^{T}-o_{i}^{T},\zeta \rangle -2s_{i1}^{o}s_{1}^{o}. \end{array}$$

We set the location of the first observatory at the origin of the earthquake coordinate system in order to simplify the equation of epicenter localization. Since the location of the first observatory $o_{1}$ is set as ${\left[0\;0\right]}^{T}$, Equation (17) can be rewritten as the following equation.

(18) $$\begin{array}{c} \bar{A}\zeta =g_{o}+p_{o}s_{1}^{o} \end{array}$$

with:

$$\begin{array}{ccc} \bar{A} & = & \left[\begin{array}{c} o_{2}^{T}\\ \vdots \\ o_{m}^{T} \end{array}\right],\;\;p_{o}=-\left[\begin{array}{c} s_{21}^{o}\\ \vdots \\ s_{m1}^{o} \end{array}\right],\;\;d=\left[\begin{array}{c} \langle o_{2},o_{2}\rangle \\ \vdots \\ \langle o_{m},o_{m}\rangle \end{array}\right],\\ g_{o} & = & \frac{1}{2}\left[\begin{array}{c} ∥o_{2}{∥}^{2}-{\left(s_{21}^{o}\right)}^{2}\\ \vdots \\ ∥o_{m}{∥}^{2}-{\left(s_{m1}^{o}\right)}^{2} \end{array}\right]=\frac{1}{2}\left(d-p_{o}•p_{o}\right). \end{array}$$

where the symbol of (•) means the Hadamard product. However, the epicenter localization formula, denoted by Equation (18), is true only under the ideal condition. In real case, there are some factors that restrict the measurement accuracy.

When we measure the P-S time to calculate the epicenter, STA/LTA algorithm depends on the user selected threshold value for earthquake location. Therefore, the obtained P-S time has a measurement error caused by the limited STA/LTA accuracy. The constrained P-S time data is closely related to finding the epicenter location using the RDOA data. As a result, the RDOA value can be represented as:

(19) $$\begin{array}{ccc} p & = & col\left\{s_{k1}^{o}+\Delta s_{k1},\;k=2,3,\cdots ,m\right\}\;\\ & = & p_{o}+\Delta p,\\ p_{o} & = & col\left\{s_{k1}^{o}\right\},\;\;\Delta p=col\left\{\Delta s_{k1}\right\},\\ E\left[\Delta p\right] & = & 0,\;\;Q_{p}=E\left[\Delta p\Delta p^{T}\right] \end{array}$$

where Δp is the epicenter inconsistency error caused by the P-S time measurement error, and col{·} means a column vector. In real environment, the parameter g is formulated using Equation (19) as follows

(20) $$\begin{array}{c} g=g_{o}+p_{o}•\Delta p. \end{array}$$

In order to express the formula of the epicenter localization under real environment including the effect of measurement error, the parameters $p_{o}$ and $g_{o}$ in Equation (18) should be replaced with p and g, respectively. Therefore, the epicenter location in real condition can be found as follows [46]:

(21) $$\begin{array}{c} \bar{A}\zeta =\bar{B} \end{array}$$

with:

(22) $$\begin{array}{ccc} \bar{B} & = & b_{o}+N\Delta p,\\ b_{o} & = & g_{o}+p_{o}s_{1}^{o}\\ N & = & diag\left(p_{o}\right)-s_{1}^{o}I \end{array}$$

The vector ζ that denotes the location of epicenter can be obtained using Equation (21). The measurement noise caused by the limited accuracy of STA/LTA method under real environment leads to the discrepancy between the estimated epicenter location and the real epicenter location. By compensating the measurement error using an optimization scheme, the precise location of epicenter (ζ) can be obtained from Equation (21).

## 5. Simulation and Experimental Results

**Figure 2:**
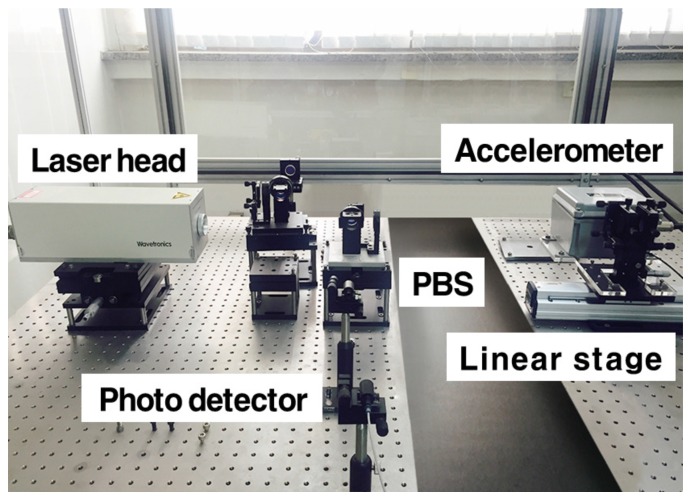
Seismic wave measurement system using a laser interferometer.

In this section, we prove the performance of the seismic signal measurement by using a laser interferometer, and obtain the normalized frequency data in the time-frequency domain using STFT algorithm. Moreover, the IF estimation, denoted by Equation (8), is applied to STFT data. Finally, we show the accurate epicenter location detection by using RDOA algorithm from the P-S time that is calculated from IF estimation through STA/LTA algorithm. Figure 2 shows the seismic wave measurement system using a heterodyne laser interferometer. The experiment uses a heterodyne laser interferometer with a He-Ne laser head (Wavetronics: WT-307B). To generate the seismic signal, we use a linear stage driven by a 2-phase stepping motor (Sciencetown: PSA6520) with a 20 mm stroke. Using the linear stage, seismic wave movement is generated to prove the performance of the interferometric seismometer. To compare the performance, we use an accelerometer as a reference since it is currently used as an earthquake motion measurement instrument. The model of JEP-8A3, Mitutoyo, is actively utilized as an acceleration sensor due to its high-performance. The measurement range, dynamic range, and sensitivity of JEP-8A3 are 3000 Gal, 145 dB, and ±3%, respectively. The displacement variation caused by the linear stage is measured by the heterodyne laser interferometer. The displacement data can be transformed to the acceleration data using a sample rate (6.7 kHz).

**Figure 3:**
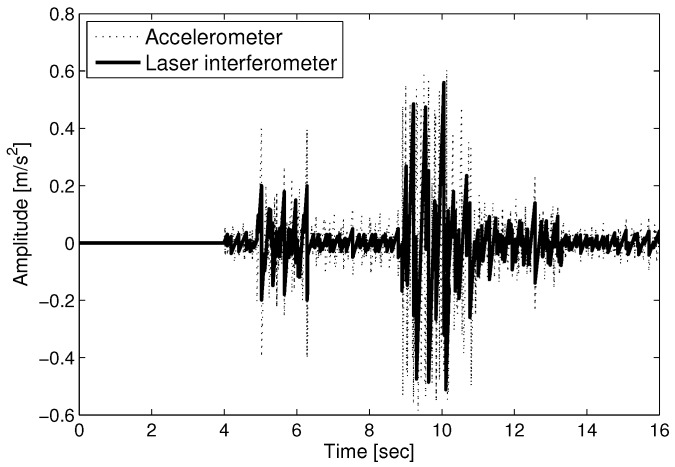
Seismic wave measurement with a laser interferometer.

We set the mean wavelength ($\lambda_{m}$) from the laser head as 632.9 (nm), and the air refractive index (n) as 1.000000026. Figure 3 shows the seismic wave movement measured by laser interferometer. The amplitude discrepancy in Figure 3 shows the sensitivity to measurement noise.

**Figure 4:**
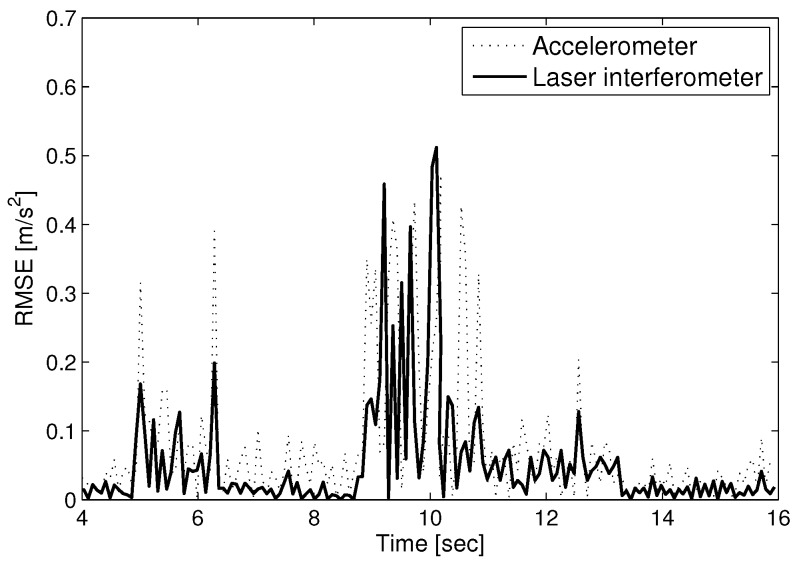
Measurement error comparison between a laser interferometer and accelerometer.

**Figure 5:**
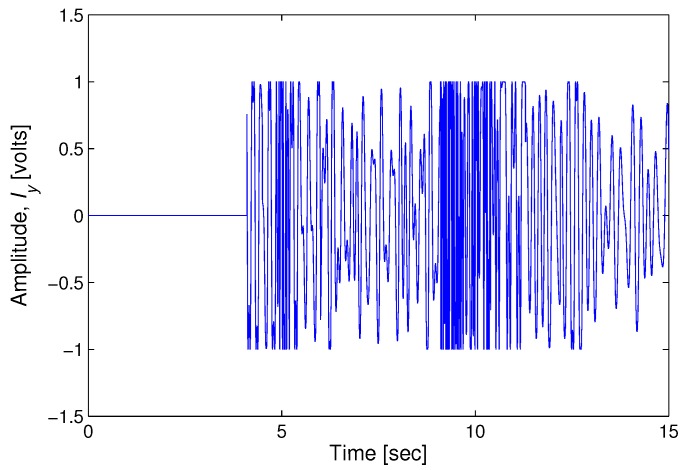
Intensity signal ($I_{y}$) for spectrum analysis.

With the displacement measurement by the heterodyne laser interferometer, the phase value (Δϕ) is proportional to the displacement (*D*), as in Equation (5). Figure 4 represents the difference between the true amplitude and the measurement value at each sampling time. The thick solid line and the thin dotted line in Figure 4 denote the root mean square error (RMSE) of the measured seismic wave using a laser interferometer and accelerometer, respectively. The measurement using a laser interferometer is more precise than the accelerometer. Figure 5 shows the intensity signal ($I_{y}$) for spectrum analysis with AB = 2 in Equation (4). The high-density points of $I_{y}$ can be interpreted as the arrival time of the P and S waves at 5 and 9 s, respectively. The amplitudes of the P and S waves changing frequently are represented by the highly dense intensities in Figure 5.

**Figure 6:**
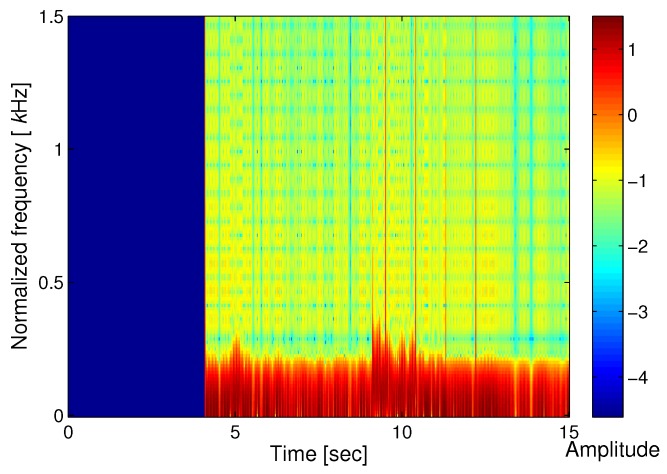
STFT result of the seismic wave.

**Figure 7:**
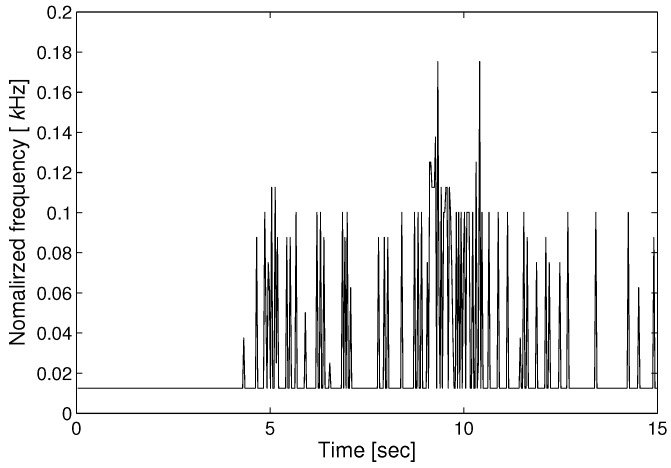
IF analysis of the seismic wave.

STFT algorithm is used to represent the detected signal in the time-frequency domain. The Fourier transform is adequate to project the data in the time domain into the frequency domain. STFT algorithm is applied to intensity signal ($I_{y}$) data that represents high density when P and S wave arrive. The differential phase value is obtained using the STFT data. Figure 6 shows the result of STFT that indicates time, frequency and amplitude distribution. The red color represents a high amplitude value, and the blue color represents a low amplitude value. The figure shows that there are sharp points at 5 and 9 s, respectively. The arrival times of the P-wave and S-wave are confirmed. After the arrival of the P and S waves, the amplitude of the seismic wave changes. Therefore, the peak points at 5 and 9 s mean the arrival time of the P and S waves. With the derived STFT data, IF estimation is performed, as shown in Figure 7. Using the result of STFT in the time-frequency domain, IF is applied to get the frequency value in each interval time. Similar to the result in Figure 6, the value in Figure 7 represents the arrival time of the seismic signal.

**Figure 8:**
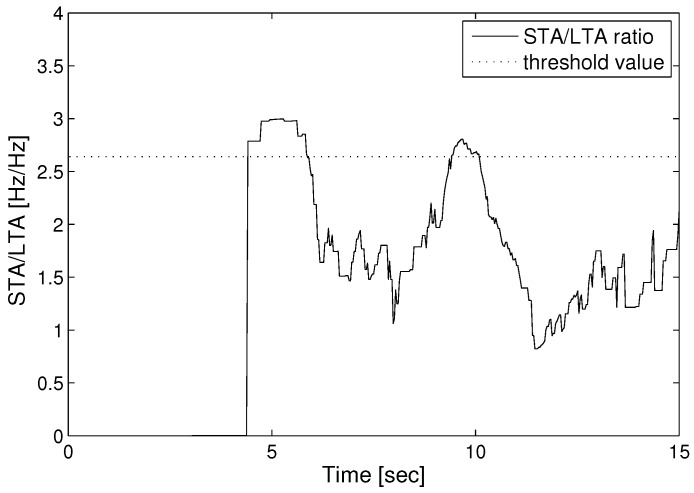
Estimation of the STA/LTA ratio.

Figure 8 shows the seismic wave data with STA/LTA algorithm. We set up the LTA window size with as much as 8 times the STA window size, and set the threshold value as twice the average of the STA/LTA ratio. We can determine the P-S time when the STA/LTA ratio becomes greater than the threshold value. The arrival times of the P-wave and S-wave were measured as 4.65 and 9.12 (s), respectively. If we suppose the velocities of the P-wave and S-wave as 8.2 and 3.7 (km/s) separately, we can calculate the distance to the epicenter as 31.2 (km), according to Equation (10).

**Figure 9:**
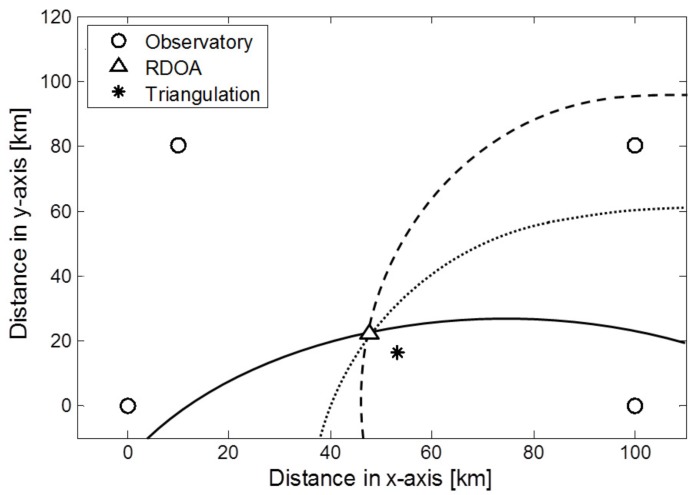
Epicenter location using RDOA algorithm.

Figure 9 shows the estimation of the epicenter location. The circle points represent the observatory locations, while the triangle point represents the epicenter location. We set the epicenter location as (50, 20) (km) when the measurement noise does not exist. We suppose the locations of the observatory as (0, 0), (10, 80), (100, 0), and (100, 80) (km), respectively. The epicenter location found using the RDOA hyperbolae is shown in Figure 9. When the hyperbolae are to be drawn, we set the observatory that is placed at (100, 0) (km) as a reference. With the reference observatory and each separate observatory, the three pairs of hyperbolae were derived. We assume that the measurement noises of epicentral distances at each observatory follow the Gaussian distribution. $n_{e}$ is the maximum absolute value of the measurement noises. We suppose the parameter $n_{e}$ as 1 in Figure 9. As a result, we determined the epicenter location using RDOA algorithm as (47.9, 21.2) (km). The asterisk point is the estimated epicenter location using triangulation method that is generally used for location estimation of an epicenter. The estimated result using the triangulation method is (53.7, 17.9) (km). It can be confirmed that the estimated result from RDOA is closer to the actual location. Table 1 shows the RMSE comparison of RDOA based estimation method with the triangulation method for various measurement noises. As the measurement noise represented as $n_{e}$ increases, the localization accuracy becomes low accordingly. It can be confirmed that the result of RDOA based epicenter estimation is better under the circumstances of measurement noise.

## 6. Conclusions

In this paper, we suggest an epicenter localization method based on RDOA algorithm. The range difference information for RDOA algorithm is obtained from a seismic signal measured by a heterodyne laser interferometer. The laser interferometer uses the Doppler effect to detect movement of the stage. We measure the seismic signal with the use of a laser interferometer. To determine the P-S time, we apply STFT, IF, and STA/LTA algorithms to seismic signal data obtained from the laser interferometer’s intensity signal ($I_{y}$). Using STFT and IF, the transformed signal of the seismic wave is obtained in the time-frequency domain. With the changes of frequency, we decide the arrival time of the P and S waves. Moreover, we determine the epicenter location with RDOA algorithm. We confirm that RDOA algorithm can more accurately estimate the epicenter location.

## Figures and Tables

**Table 1:** RMSE comparison for various measurement noises ($n_{e}$).

Methods	RMSE (km)				
	$n_{e}$ = 1	$n_{e}$ = 2	$n_{e}$ = 3	$n_{e}$ = 5	$n_{e}$ = 10
Triangulation	4.25	6.12	7.28	8.92	13.29
RDOA	2.41	3.29	3.93	4.72	7.20
